# Selective distant electrostimulation by synchronized bipolar nanosecond pulses

**DOI:** 10.1038/s41598-019-49664-2

**Published:** 2019-09-11

**Authors:** Elena C. Gianulis, Maura Casciola, Carol Zhou, Enbo Yang, Shu Xiao, Andrei G. Pakhomov

**Affiliations:** 1 Frank Reidy Research Center for Bioelectrics, Old Dominion University, Norfolk, VA 23508 USA; 2 Department of Electrical and Computer Engineering, Old Dominion University, Norfolk, VA 23508 USA

**Keywords:** Biophysical methods, Biological fluorescence

## Abstract

A unique aspect of electrostimulation (ES) with nanosecond electric pulses (nsEP) is the inhibition of effects when the polarity is reversed. This bipolar cancellation feature makes bipolar nsEP less efficient at biostimulation than unipolar nsEP. We propose to minimize stimulation near pulse-delivering electrodes by applying bipolar nsEP, whereas the superposition of two phase-shifted bipolar nsEP from two independent sources yields a biologically-effective unipolar pulse remotely. This is accomplished by electrical compensation of all nsEP phases except the first one, resulting in the restoration of stimulation efficiency due to cancellation of bipolar cancellation (CANCAN-ES). We experimentally proved the CANCAN-ES paradigm by measuring YO-PRO-1 dye uptake in CHO-K1 cells which were permeabilized by multiphasic nsEP (600 ns per phase) from two generators; these nsEP were synchronized either to overlap into a unipolar pulse remotely from electrodes (CANCAN), or not to overlap (control). Enhancement of YO-PRO-1 entry due to CANCAN was observed in all sets of experiments and reached ~3-fold in the center of the gap between electrodes, exactly where the unipolar pulse was formed, and equaled the degree of bipolar cancellation. CANCAN-ES is promising for non-invasive deep tissue stimulation, either alone or combined with other remote stimulation techniques to improve targeting.

## Introduction

Electrical stimulation (ES) is one of the most universal approaches to manipulate biological functions. Effects of ES are diverse and range from nerve and muscle excitation, Ca^2+^ mobilization, activation of immune and endocrine systems, tissue differentiation and regeneration, to membrane permeabilization and initiation of cell death. ES has many established clinical applications, including cardiac pacing^1,2^, defibrillation^3,4^, muscle training and rehabilitation^5,6^, pain control^7–10^, treatment of neuromuscular, psychiatric, and neurodegenerative diseases^11–17^, and cancer ablation^18,19^.

ES is accomplished either invasively, by the insertion or implantation of electrodes, or non-invasively from the surface. Non-invasive ES modalities such as transcutaneous electrical nerve stimulation^9,20–22^ and transcranial direct current stimulation^9^ deliver the electric field indiscriminately to the tissue volume between electrodes. The transcranial magnetic stimulation (TMS) is a non-invasive procedure that became a breakthrough in neural stimulation and therapies^17,23–27^, but with its own limitations including limited penetration^28,29^ and challenges in targeting^28^.

The accepted way to target ES precisely to a specific area within the brain or body is by invasive techniques with inserted or implanted electrodes. Tissue damage, pain, risks of bleeding, infection, and inflammation associated with electrode placement preclude the use of invasive techniques for routine examination of patients, disease diagnostics, and for treatments which do not justify implantation surgery (such as outpatient treatment of drug addiction by brain stimulation^15–17^).

The electric field is generally higher near the electrodes and decays with distance^30–32^, hence the excitation and tissue damage in the immediate vicinity of electrodes occur at a lower applied voltage (current) than at a distance. Therefore, it is challenging to achieve selective stimulation at a distance while avoiding a response near the electrodes, such as to selectively stimulate deep tissue from surface electrodes. Several studies attempted achieving deep penetration along with improved spatial precision of ES by modifying the shape, configuration, and/or number of electrodes or magnetic stimulation coils. The invention of H-coils has extended the depth of TMS from usual 1.5–2 cm to 4–6 cm^28,33^, but at the expense of wider electric field exposure and poor targeting. Out of various disc and ring electrode configurations for transcranial electrostimulation, the concentric ring configuration, which comprises a cathode ring surrounding an anode disc electrode, provided the highest spatial focality, although at the expense of increased current demand and poor penetration depth^30^. In spite of its drawbacks, the concentric ring was subsequently developed into a 4 × 1 ring electrode array, with 4 cathode electrodes surrounding a single anode electrode, and has been employed for high-definition, targeted neurostimulation^31,34–36^.

Recent breakthrough studies introduced two original approaches for targeted remote ES, namely the Intersectional Short Pulse (ISP) stimulation^37^, and stimulation by temporal interference (TI) of two sine waves with slightly different frequencies^38^. The ISP approach relies on temporal summation of excitatory effects of short pulses delivered with short delays, in a rotating manner, by several pairs of electrodes. The maximum summation and, expectedly, the maximum response is evoked at the intersection of lines connecting electrodes in each pair. By varying the position of electrodes, the maximum stimulation can be focused within the left or right hemisphere, with only minor effects on the surface of the head. The TI stimulation approach relies on the lack of neuronal response to a continuous high-frequency sine wave signal (1 kHz or higher). Delivering two such sine waves with a small frequency shift (e.g., 1 kHz and 1.01 kHz) causes a low-frequency amplitude modulation of the overlapping sine waves remotely from the electrodes. This signal is somehow demodulated by neurons, resulting in their repeated firing at the modulation frequency. Both the ISP and TI approaches require remarkably low electric field, on the order of 1–4 mV/mm, to elicit neuronal response in the target area. The exact biophysical mechanism of how such weak electric field is detected by brain tissue to trigger action potentials remains to be elucidated.

Here we introduce a new paradigm of focused remote stimulation based on bipolar cancellation. This term stands for a unique property of ultra-short electric stimuli to cancel their effects when the pulse polarity is reversed. This phenomenon was originally described for the activation of peripheral nerve fibers, where the addition of the opposite polarity phase to stimuli tens of µs long suppressed the action potential (AP) and/or increased its threshold^39–42^. Fast reversal of the electric field halted the process of opening of voltage-gated Na^+^ channels, which takes no less than approximately 10 µs^43^. With recent expansion of ES studies to still shorter, nanosecond-range electric pulses (nsEP), the bipolar cancellation was established universally for diverse ES effects, from Ca^2+^ mobilization in excitable and non-excitable cells, to membrane permeabilization and cell death induction, and may engage biophysical mechanisms other than channel opening^44–49^.

**Figure 1:**
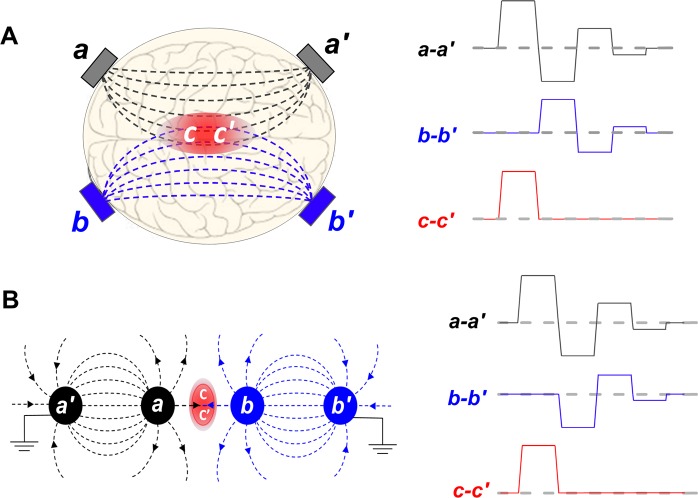
Targeted remote stimulation by superposition of two bipolar nanosecond pulses into a unipolar stimulus. The approach is illustrated for a rectangular (**A**) and linear (**B**) arrays of stimulating electrodes. In (**A**), two pairs of independent, ground-isolated electrodes (*a-a*′ and *b-b*′) deliver two synchronized bipolar electric pulses such as shown in the right panel. Each of the bipolar pulses is inherently inefficient for electrostimulation due to bipolar cancellation (see text), but their superposition in *c-c*′ area forms locally a biologically-effective unipolar pulse. In (**B**), the formation of a unipolar pulse in the *c-c*′ area is accomplished by bringing the electrode *b* to the same electric potential as electrode *a* during the 2^nd^ and the 3^rd^ phases of the pulse. See text and Fig. 2 for more detail.

Figure 1A illustrates how the bipolar cancellation can be employed for stimulation of an area distant from electrodes using a **can**cellation of **can**cellation (CANCAN) paradigm. A bipolar nsEP applied to either *a-a*′ or *b-b*′ pair of electrodes is inherently inefficient for stimulation and will cause little response. However, superposition of two properly shaped and synchronized bipolar nsEP into a unipolar stimulus should cancel the cancellation imposed by the bipolarity and restore the ES efficiency in *c-c*′ area, remotely from the electrodes.

**Figure 2:**
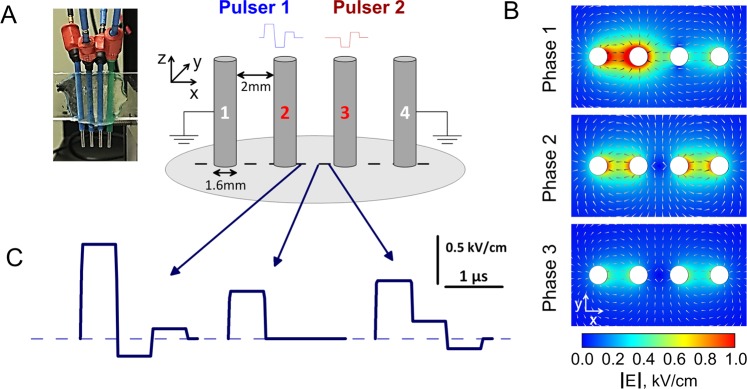
Linear electrode array for testing CANCAN stimulation paradigm. (**A**) a photo and a schematic of the array, which consists of 4 hollow electrodes (1.6-mm outer diameter, 2 mm separation). The electrodes pierce agarose gel containing seeded cells (gray oval, not to scale). Two central electrodes deliver synchronized bipolar stimulating pulses (600 ns each phase). Pulse to electrode 3 is delayed by one phase (“phase-shifted” delivery) and copies phases 2 and 3 of the pulse at electrode 2, as in Fig. 1B. (**B**) Electric field in the gel in the XY plane (perpendicular to the electrodes) during 3 phases of the pulse. Note cancellation of the electric field in the center of the array during the 2^nd^ and 3^rd^ phases when electrodes 2 and 3 are equipotential. (**C**) Superposition of the bipolar pulses yields a unipolar pulse in the center of the gap between electrodes 2 and 3 and complex waveforms in the proximity of the electrodes (see Fig. 3 for more detail). Simulations for panels (B,C) assumed the amplitude of 200, 140, and 80 V for pulse phases 1–3, respectively (100/70/40% ratio).

**Figure 3:**
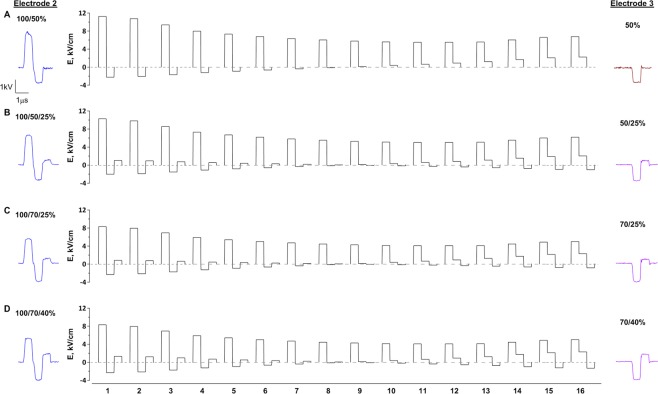
Stimulus shapes formed by the superposition of bipolar pulses along the line between two active electrodes. The distance between electrodes 2 and 3 (see Fig. 2A) was split into 16 equal segments which correspond to the regions of interest for fluorescence measurements. Rows (**A***–***D)** correspond to different stimulation conditions employed for experiments presented in Figs 4 and 5. Pulses delivered to electrodes 2 and 3 (actual oscilloscope traces) and their phase ratios are shown in each row in the left and right panels, respectively (the first phase delivered to electrode 2 is taken as 100%). Pulses delivered to electrode 3 omit the first phase and start with a 600 ns delay, as explained in Figs 1B and 2. Electric field simulations for the middle panels assumed idealized (rectangular) shape of all phases of delivered pulses.

For this study, which goal was to get a proof-of-concept validation of the CANCAN paradigm, we chose to use a linear electrode array (Figs 1B and 2). This is a simpler electrode configuration, which enables measurements of the CANCAN effect in just a single dimension, along the line connecting two central electrodes. These active electrodes delivered two identical nsEP, except for the omission of the first phase in one of them (Fig. 1B). The electric field in the center of the array (the middle point between two active electrodes) was present during the first phase of nsEP but got compensated during the subsequent phases, when the active electrodes were brought to the same potential (Fig. 2C). However, a “clean” unipolar pulse was formed only in the center of the array, whereas the incomplete compensation at other locations resulted in various complex waveforms (Fig. 3), with unknown bipolar cancellation capacity. CANCAN-ES requires that these complex waveforms exert bipolar cancellation which diminishes from the electrodes to the center of the array. By just a visual assessment, the “bipolarity” of these complex waveforms decreases from the periphery to the center, suggesting a concurrent weakening of bipolar cancellation. To validate this conjecture, we needed to measure ES efficiency at multiple locations along the line between the electrodes. Such measurements would be difficult to perform in real time; therefore we chose membrane electroporation and dye uptake by permeabilized cells as a long-lasting and reliable endpoint of ES efficiency. Despite a low degree of bipolar cancellation for electroporation (typically, 3-5-fold^45,47^), our experiments demonstrated a highly reproducible enhancement of the effect due to CANCAN-ES remotely from stimulating electrodes.

## Results

### Electropermeabilization and bipolar cancellation in gel-embedded CHO cells

**Figure 4:**
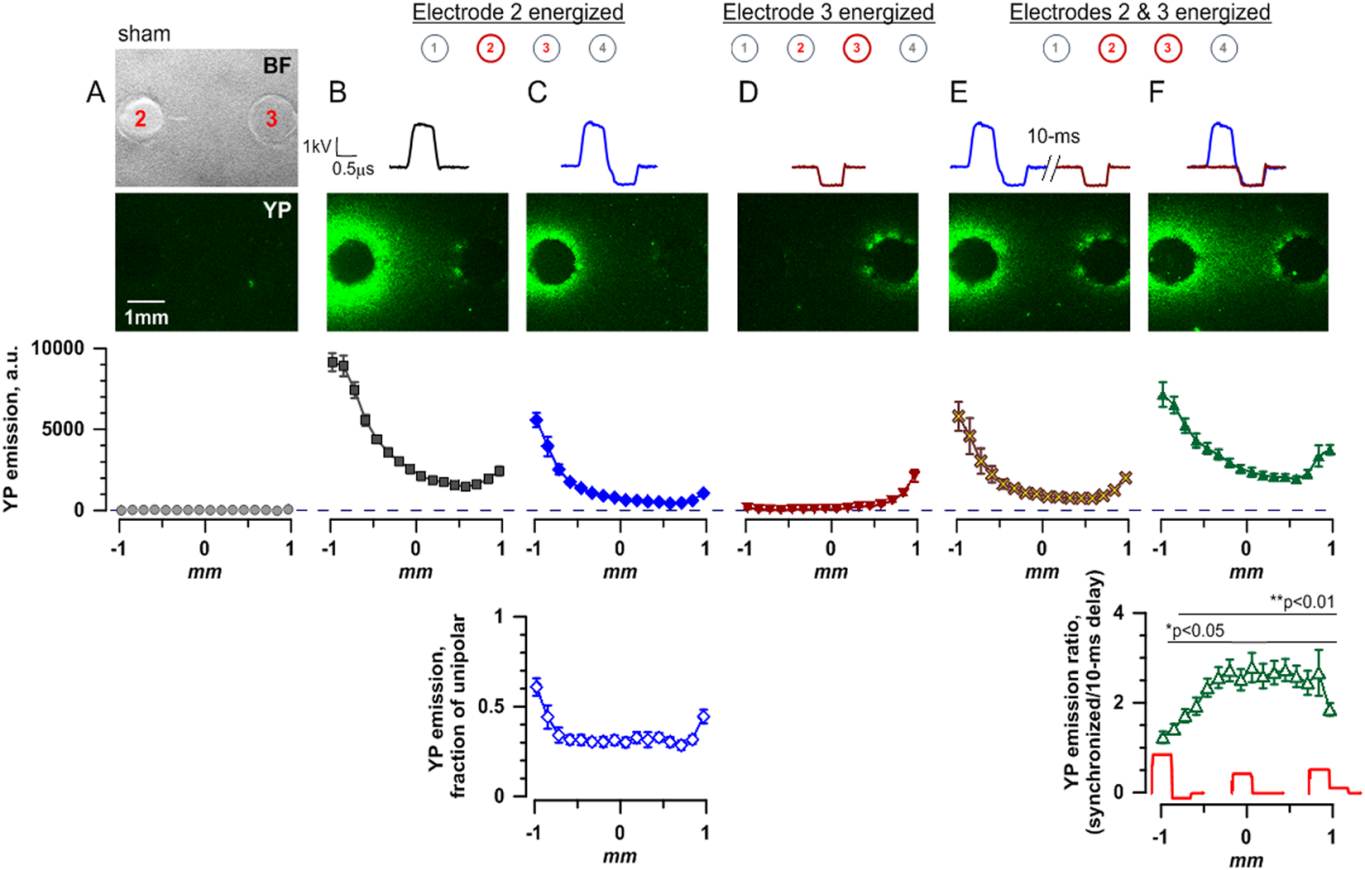
Enhancement of stimulation away from electrodes by the abolition of bipolar cancellation. Stimulation effect was quantified by the uptake of YO-PRO-1 dye (YP) by electropermeabilized cells embedded in agarose gel. Panels (A–F) correspond to different stimulation conditions. For (**B–F**), we delivered 100 stimuli at 10 Hz; the legend at the top shows the electrode(s) energized and the shape of the pulse. In (**A**), stimulating electrodes were brought into position but no pulses were applied (“sham exposure”); the top panel shows instead the gel imprints of the electrodes 2 and 3 (see Fig. 2 for the linear array configuration). Areas beyond this region are not considered here (see text and Fig. S1). All YP fluorescence images cover the same area as in the bright field image in panel (A). Below these images, fluorescence intensity measured in 16 regions of interest along the line between the electrodes 2 and 3 is plotted against the distance from the center of the gap between them (mean ± S.E., *n* = 5). For all exposures, dye uptake is higher near the energized electrode(s) where the electric field is the highest. Note the reduction of effect in panel (C) vs (B), when an opposite polarity phase is added to the unipolar pulse. This bipolar cancellation is quantified as a reduction of emission compared to the unipolar pulse (**C**, bottom graph), which was significant at p < 0.01 or better for all datapoints (one sample *t*-test, for the ratio being different from 1). In panels (E,F), we combine the same pulses which were tested separately in panels (C,D). When the pulses are delivered with a 10-ms interval (**E**) which prevents superposition and pulse shape change, their combined effect is essentially additive. However, when the same two pulses overlap (**F**) so that the opposite polarity phase is reduced or fully compensated, bipolar cancellation gets reduced and the effect increases up to 3-fold (panel (F), bottom; the inset illustrates some of the pulse shapes formed by the overlap of delivered pulses). *p < 0.5, **p < 0.01, one sample *t*-test.

We first had to establish nsEP exposure conditions to cause measurable YP uptake while avoiding signal saturation, for any nsEP shape and for all locations along the line between active electrodes 2 and 3 (Fig. 2A). These measurements had to encompass YP uptake variations due to a combined impact of nearly a 2.5-times electric field change from the electrode edge to the middle point of the array (Figs 2 and 3), about 2-fold difference in the peak amplitudes of nsEP from Pulsers 1 and 2 (Fig. 3), and 2- to 5-fold reduction of the effect by bipolar cancellation^47^. We have identified exposure parameters which enabled reliable measurements under all of the above conditions (100 pulses, 600-ns duration of each phase, 10 Hz, 1.7–2.3 kV peak amplitude from Pulser 1, Figs 4, S1 and S2). The pulse duration, number, and repetition rate were kept constant throughout the study, whereas the peak amplitude, the number of phases, and phase amplitude ratios were varied as outlined for each experiment. The 2^nd^ and 3^rd^ phases, when present, could be set to 50–70% and 25–40% of the first phase, respectively, with the resulting phase ratios such as 100/50/25% and 100/70/40%. For CANCAN-ES, Pulser 2 generated nsEP which skipped the first phase and matched phases 2 and 3 of the nsEP from Pulser 1 (Fig. 1B). The employed exposure parameters could cause saturation of the response between electrodes 1 and 2 (Fig. S1), but it was outside the area of testing for CANCAN-ES and was thus disregarded.

### Compensation of the opposite polarity phase restores nsEP efficiency

Figure 4 shows typical patterns of YP uptake in the region between electrodes 2 and 3 following different nsEP treatments. Sham exposures (insertion of electrodes without nsEP delivery) caused no considerable dye uptake in either this set of experiments (Fig. 4A) or in any subsequent series (data not shown). Unipolar 2.3 kV pulses applied to electrode 2 (Fig. 4B) expectedly caused maximum dye uptake near the electrode; the effect decayed with distance, following closely the electric field distribution (Figs 2B and S4). The addition of a 1.2 kV 2^nd^ phase of the opposite polarity caused robust bipolar cancellation, reducing the response 2-3-fold (p < 0.01; Fig. 4C). The cancellation was stronger away from the electrodes (lower panel). A unipolar 1.2 kV pulse applied to electrode 3 permeabilized cells mostly near the electrode, with much weaker response at a distance (Fig. 4D). When electrodes 2 and 3 were energized with a 10-ms delay, which excluded a change in the pulse shape due to compensation of the 2^nd^ phase, the resulting effect was simply additive (Fig. 4E). However, when the same nsEP were delivered without a delay, the effect increased more than twofold in the region where the 2^nd^ phase of the pulse was compensated (p < 0.01; Fig. 4F; also see Fig. 3A for local electric field intensities and waveforms). The effect at the middle point between the electrodes was the same as from the unipolar pulse in Fig. 4B. Thus, we were able to reduce the electric field effect near the electrode 2 while preserving it at a remote location, which is exactly the goal of CANCAN-ES. Since the bipolar pulse was delivered to electrode 2 only, the attenuation of the effect expectedly did not occur near electrode 3. Instead, the superposition of two pulses near electrode 3 added a small 2^nd^ phase of the same polarity (inset in Fig. 4F, lower panel, and Fig. 3A), which slightly increased the effect at this location. To overcome this deficiency, below we combined triphasic pulses applied to electrode 2 with biphasic pulses applied to electrode 3. Noteworthy, the enhancement of the effect due to the CANCAN effect was approximately equal to the degree of bipolar cancellation (compare lower panels in Fig. 4C,F).

### Synchronization of multiphasic nsEP improves CANCAN-ES targeting

We reduced the amplitude of the 1^st^ and 2^nd^ phases to 2.1 and 1.1 kV, respectively, since the data in Fig. 4C suggested better bipolar cancellation at lower electric field strengths, i.e. farther away from the electrodes. Concurrently, we added a 0.4-kV 3^rd^ phase to nsEP from Pulser 1 and an identical 2^nd^ phase to nsEP from Pulser 2. Figure 3B shows the variety of the electric field waveforms produced by the superposition of these two nsEP.

**Figure 5:**
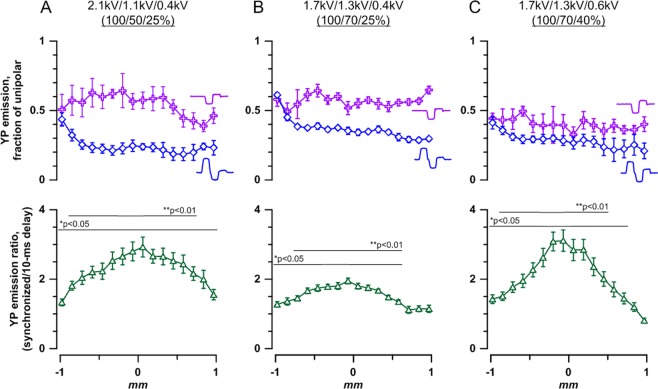
Stronger bipolar cancellation rendered by multiphasic nsEP improves stimulation targeting. Cell electropermeabilization assay and stimulation conditions are the same as described in Fig. 4. For panels (A–C), the shapes of stimulating pulses and local waveforms arising from their superposition are provided in Fig. 3, (**B–D)**, respectively. The amplitude of each phase (kV) and their ratio (%) for a triphasic nsEP applied to electrode 2 are also given at the top of each panel; biphasic pulses applied to electrode 3 are the same but omit the first phase. Top panels show the degree of bipolar cancellation when additional phase(s) are added to a unipolar pulse. The data plots for tri- and biphasic pulses are identified by respective symbols. YP emission caused by stimulation with tri- and biphasic pulses is plotted as a fraction of the effect of the respective unipolar pulse at the same location. Abscissa is the distance from the center of the gap between electrodes 2 and 3 along a line connecting them (Fig. 2A). See Fig. S2 for YP emission values without normalization to unipolar pulse data. Bipolar cancellation is significant at p < 0.05 or better for all datapoints (one sample *t*-test, for the ratio being different from 1). Bottom panels: The enhancement of effect remotely from the electrodes when the tri- and bi-polar pulses are delivered synchronously (with one phase shift) to electrodes 2 and 3 respectively. For comparison, the same pulses are delivered with a 10 ms delay, to prevent their overlap and opposite polarity compensation (same as in Fig. 4F). See Fig. S2 for actual values of the effects of these pulse delivery protocols. Mean ± S.E., n = 5–6. *p < 0.5, **p < 0.01, one sample *t*-test.

The effects of uni- and bipolar nsEP (with either 3 or 2 phases), when applied separately to electrode 2 or 3, respectively (Fig. S2A, top and middle panels), were essentially the same as described in the previous section and anticipated from the electric field distribution. The triphasic nsEP caused strong cancellation of the response, reducing the response 2-fold near the electrode and 5-fold at a distance (Figs 5A and S2A, p < 0.01 for all datapoints). The biphasic pulse caused overall weaker cancellation, with approximately twofold effect reduction compared to the respective unipolar pulse (Figs 5A and S2A, p < 0.05 for datapoints in the vicinity of the electrode; farther away, the effect is weak and the ratio becomes too noisy). Synchronized delivery of both bipolar nsEP to electrodes 2 and 3 compensated the 2^nd^ and the 3^rd^ phases in the middle of the gap between the electrodes (Fig. 3B), increasing the electroporation 3-fold compared to the effect of the same pulses delivered with a 10-ms delay (p < 0.01, bottom panels in Figs 5A and S2A). Thus, the employed combination of multiphasic nsEP enabled the reduction of stimulation near both electrodes and maximizing the effect remotely from them. Remarkably, all complex waveforms which formed between the electrodes (Fig. 3B) caused bipolar cancellation which weakened towards the center between the electrodes. This feature followed the visual perception of the pulse “bipolarity,” thereby prompting first attempts to quantify it and link to the efficiency of bipolar cancellation (see Supplementary Information).

We further tried to tweak the nsEP treatment parameters in order to achieve stronger bipolar cancellation and improve stimulation targeting. Since cancellation by the triphasic pulse was stronger away from the active electrode, where the electric field was weaker (Fig. 5A, top panel), we reduced the first phase from 2.1 to 1.7 kV to lower the electric field throughout the target area. On the contrary, the biphasic nsEP produced more cancellation close to the active electrode, in the region where the electric field was stronger (Fig. 5A, top panel); therefore, we increased the 2^nd^ phase from 1.1 to 1.3 kV. However, a combination of these two changes had the opposite effect, reducing cancellation by the 3-phase pulse (although still significant at p < 0.01), and worsened stimulation targeting when both bipolar nsEP were combined (p < 0.01 in the center; Figs 5B and S2B). The situation was remedied by increasing the amplitude of the 3^rd^ phase from 0.4 to 0.6 kV, which enhanced cancellation by both tri- and biphasic pulses and resulted in the best stimulation targeting (p < 0.01 in the center, Figs 5C and S2C).

## Discussion

This study presents the first proof-of-concept validation of the CANCAN concept for electrostimulation of deep targets remotely from electrodes. This is accomplished by the change of the stimulating pulse shape within the cell culture or tissue volume between stimulating electrodes – from the unipolar shape in the middle of the gap between the electrodes, to biologically-inefficient bipolar shape immediately next to the electrodes. This gradual loss of stimulation efficiency due to the increase of pulse “bipolarity” (and, consequently, stronger bipolar cancellation) offsets the increase of the stimulation efficiency due to gradual strengthening of the electric field close to the electrodes.

Obviously, stronger bipolar cancellation can overcome larger gradients of the electric field, resulting in more targeted stimulation at a larger distance from electrodes where the pulse shape is unipolar or close to unipolar. As explained in the Introduction, for this study we deliberately chose electroporative YP dye uptake as an endpoint to demonstrate the CANCAN effect, despite relatively weak bipolar cancelation (3-4-fold). The advantage of choosing this endpoint was in high-resolution, stable measurements of the effect in multiple regions between the electrodes, which has made it possible to observe and measure the CANCAN-ES effect reproducibly in multiple series of experiments. While we could not eliminate electroporation near the electrodes with this weak bipolar cancellation, the gradient of the effect with distance from the electrodes was much less steep with CANCAN effect than without it.

For other endpoints, such as peripheral nerve stimulation, we consistently observe more than a 10-fold difference in stimulation thresholds for uni- and bipolar 300-ns stimuli^43^, which is in excellent agreement with predictions of SENN model of neurostimulation^50,51^. The extension of the model predictions^50^ down to 10-ns pulse duration yields almost a 100-fold threshold difference. Stronger bipolar cancellation means that we can achieve CANCAN stimulation remotely while still avoiding excitation by stronger electric field near the electrodes. Nerve fibers can be excited by 12-ns stimuli thousands of times with no damage^52^, making them perhaps the most promising target for a distant CANCAN-ES while avoiding any stimulation near the electrodes. Profound differences (5- to 20-fold) in cytosolic Ca^2+^ activation by uni- and bipolar nsEP of different durations^44^ extend potential CANCAN-ES applications to the multitude of biological processes which involve Ca^2+^ signaling^53^.

In contrast to other distant stimulation methods which attempt to enhance the effect of the applied electric field at a remote location^37,38^, CANCAN-ES relies on weakening of the electric field effects in the vicinity of the electrodes. Notably, the electric field remains most intense near the electrodes (which is a fundamental physical principle) but we reduce its biological effectiveness by making pulses “more bipolar” closer to electrodes. The maximum effect at the remote location, by definition, has the same magnitude as is achieved simply by applying a unipolar pulse from one pair of electrodes, which is consistent with experimental observation (Figs 4F and S2, bottom panels), whereas bipolar cancellation weakens or prevents the effect near the electrodes. This opens an opportunity to combine CANCAN-ES with other methods such as ISP^37^ to take advantage of both methods and achieve the most selective remote targeting. In this example, ISP stimuli produced by the superposition of bipolar stimuli (rather than directly by applying voltage between two electrodes) will offer the same focusing of the stimulus on target as ISP alone, plus the reduction of the effect near the electrodes as achieved with CANCAN-ES alone.

Moreover, the integration and temporal summation of cell membrane potential shifts from multiple nsEP delivered as high repetition rate bursts can remove the need for high voltages and strong electric fields which are generally required for nsEP stimulation. Such reduction of the electric field would be particularly useful for picosecond pulse stimulation, which is considered for localized deep brain stimulation with broadband antennas^54–56^, without any electrodes. The targeting of the stimuli improves with shortening of the pulse, but at the expense of prohibitively high stimulation voltages and bipolarity (which is inherent for emitted pulses). A high-rate CANCAN-ES can overcome or offset both of these challenges.

In summary, the CANCAN-ES paradigm offers a new way of targeted remote stimulation, which can be employed either on its own or in combination with other approaches based on different principles.

## Materials and Methods

### Cell line and media

Chinese hamster ovary (CHO-K1) cells were purchased from the American Type Culture Collection (ATCC, Manassas, VA). Cells were cultured in F-12K medium (Mediatech Cellgro, Herndon, VA) supplemented with 10% fetal bovine serum (Atlanta Biologicals, Flowery Branch, GA), 100 IU/mL penicillin, and 0.1 μg/mL streptomycin (Gibco Laboratories, Gaithersburg, MD). This medium with the additives is hereinafter referred to as “F-12K complete” medium.

### Three-dimensional cell culture

On the day of experiments, cells were embedded in an agarose gel three-dimensional (3D) culture, similar to previously described methods^57^. The bottom of a 60 mm dish was coated with 7 mL of 2% low-gelling-temperature agarose (Sigma-Aldrich, St. Louis, MO) in F-12K complete medium. Cells were harvested and resuspended in 0.75% agarose in the F-12K complete medium at 5 × 10^6^ cells/mL; 4 mL of this suspension was deposited dropwise over the 2% agarose base layer in a 60 mm dish. The dishes were incubated at 4 °C for 5 minutes to hasten agarose jellification and prevent cell sedimentation, and then transferred to the incubator for at least 30 minutes before nsEP exposure. YO-PRO-1 iodide (YP; 1 µM in 3 mL PBS; Thermo Fisher Scientific, Waltham, MA) was added to each dish 5 minutes prior to nsEP exposures and incubated at 37 °C to allow the dye to equilibrate throughout the agarose gel.

### Electrodes and nsEP exposures

nsEP delivery to cells embedded in agarose 3D cultures was accomplished using two pairs of stainless steel needle electrodes arranged in a linear array that were each connected to an independent nsEP-delivering Pulser (Fig. 2A). The two middle electrodes were active, and the two on the periphery were ground. The linear array was mounted on a micromanipulator to enable accurate and steady insertion of the electrodes into an agarose gel containing cells.

Currently, there are no commercially available bipolar pulse generators which provide sufficiently high power needed for CANCAN-ES. Therefore, in this study, we used a custom-built multiphasic pulse generator, as recently described^58,59^. nsEP were produced using a combination of three separate MOSFET-based pulse generators that were each capable of producing a uniphasic or biphasic nsEP, and two separate high-voltage DC power supplies. Each generator consisted of two stacks of fundamental modules containing a charging capacitor and a MOSFET switch, which produced either a positive or negative pulse to the desired voltage. Two of the generators were combined (Pulser 1) to deliver either a uniphasic, biphasic or triphasic nsEP to electrode 2, while the third generator (Pulser 2) delivered either a uniphasic or biphasic pulse to electrode 3. A digital delay generator (model 577-8C, Berkeley Nucleonics Corporation, San Rafael, CA) was used to control the pulse duration (600 ns for each phase), the number of phases, and the delay between them. The exact shape and amplitude of the nsEP were monitored using a Hantek DSO5202P oscilloscope (Qingdao, Shandong Province, China). For simplicity, the reported phase amplitude ratios are given as a percentage of the first phase.

In each experiment, cells were exposed to 100 pulses (delivered at 10 Hz). The pulse duration, number, and repetition rate were kept constant throughout the study; the number of phases, the peak amplitude of the applied voltage (ranging from 1.7–2.3 kV), and phase amplitude ratios were varied and are outlined for each experiment. The electric field distribution between electrodes 2 and 3 for the various peak first phase amplitudes tested is shown in Fig. S4. For accurate comparison, all nsEP and sham (no nsEP delivered) exposures were performed in a random order in the same cell sample, with up to 8 exposures per 60 mm dish. All nsEP exposures were conducted at room temperature (22 ± 2 °C).

### nsEP dosimetry and CANCAN modeling

A 3D model of the 4 electrode linear array was implemented using the commercial finite element method solver COMSOL Multiphysics®, Release 5.0 (COMSOL Inc., Stockholm, Sweden). See the Supporting Information for details on the model.

To model the delivery of the first phase, 1 V was applied to electrode 2, and electrode 3 was disconnected from the circuit. Electrodes 1 and 4 were set to 0 V. The E field produced was then scaled according to the amplitude of the subsequent phases.

CANCAN-ES takes advantage of the spatial superposition of two multiphasic pulses: the electric field produced by the two pairs of electrodes sum up for vector components with the same direction, while subtract when opposite, producing a unipolar exposure in a region distant from the electrodes, and bipolar elsewhere. Figure 2B shows the |**E**| distribution during each phase of the 100/70/40% exposure in the plane perpendicular to the electrodes at 3.8 mm from the petri dish bottom, i.e. in correspondence to the layer of cells. When the first phase (Fig. 2B, top panel) was delivered the |**E**| was higher between electrodes 1 and 2 and decayed towards electrode 4. When both Pulsers delivered an electric voltage of same amplitude and polarity (Fig. 2B, second and third phases), the subtraction of the **E** components of opposite direction produced a reduction of |**E**| in the area between electrodes 2 and 3. The y component of the electric field between electrodes 2 and 3 was ~0 kV/cm. The **E**_**x**_ was extracted as a function of time for 16 regions of interest between electrodes 2 and 3 (Figs 2C and 3). Figure 2C shows that **E**_**x**_ was maximum during the first phase (0–600 ns). During the subsequent phases (600–1800 ns), **E**_**x**_ was completely abolished (0 kV/cm) only at the center between electrodes 2 and 3, resulting in a unipolar pulse. Whereas, near the edges of the electrodes the polarity of the pulse changed, targeting the areas of possible bipolar cancellation. See Supporting Information for a detailed quantification of the change in polarity (Fig. S3).

### Cell imaging and data processing

After nsEP exposures, dishes were kept covered for 15 minutes, and then washed 5 times with PBS to remove all YP. Images of electropermeabilized cells were acquired using an Olympus SZX16 fluorescence stereo microscope (Olympus America, Hamden, CT) equipped with a Hamamatsu C9100 EM-CCD camera (Hamamatsu, Shizuoka Prefecture, Japan) and a 0.8x, 0.12 NA objective. YP emission was detected using an X-Cite Series 120Q fluorescence light source (Excelitas Technologies Corporation, Waltham, MA) and a GFP filter (ex. 460–490 nm/em. 510 nm longpass).

Images were quantified using MetaMorph 7.8.13 software (Molecular Devices, Foster City, CA). The background-corrected YP fluorescence was measured within 16 regions of interest along a long drawn between electrodes 2 and 3 (Fig. 2A) and plotted against distance from the center of the gap between the electrodes.

### Statistical analysis

Data are presented as mean ± S.E for *n* independent experiments. Statistical analyses were performed using a Student’s *t*-test, as either a two-tailed *t*-test (for comparison of two independent groups; Fig. S2) or one-sample *t*-test (for data presented as a ratio, with the ratio being different from 1; Figs 4 and 5). *P* < 0.05 was considered statistically significant.

## Supplementary information

Supplementary Information for Selective distant electrostimulation by synchronized bipolar nanosecond pulse

## Data Availability

All data generated or analyzed during this study are available from the corresponding author on reasonable request.
