# Supplementary material for: Selective distant electrostimulation by synchronized bipolar nanosecond pulses
Source: Sci Rep. 2019 Sep 11;9:13116. doi: 10.1038/s41598-019-49664-2 (PMC6739416; doi:10.1038/s41598-019-49664-2)
Supplement: Supplementary file 1 — Supplementary Information for Selective distant electrostimulation by synchronized bipolar nanosecond pulse [file 41598_2019_49664_MOESM1_ESM.pdf]

Supplementary Information for

Selective distant electrostimulation by synchronized bipolar nanosecond pulses

Elena C. Gianulis[a], Maura Casciola[a], Carol Zhou[a], Enbo Yang[a], Shu Xiao[a,b], and Andrei G. Pakhomov[a*]

[a]*Frank Reidy Research Center for Bioelectrics, Old Dominion University, Norfolk, VA 23508, USA*

[b]*Department of Electrical and Computer Engineering, Old Dominion University, Norfolk, VA 23508, USA*

***Corresponding author:**

Andrei G. Pakhomov

Frank Reidy Research Center for Bioelectrics,

Old Dominion University

4211 Monarch Way, Suite 300, Norfolk, VA 23508

(210) 204-9012, (757) 683-8003

Fax: (757) 451-1010

2andrei@pakhomov.net

Model details for nsEP dosimetry and CANCAN modeling

The configuration used for electric field simulations included two pairs of stainless steel needle electrodes (1.66 mm diameter, 3 cm height), which were arranged in a linear array (2 mm inter-electrode distance; Fig. 2A). The electrodes were immersed in a solution with conductivity of 1.4 S/m and relative permittivity of 76. They were positioned 1 mm above the bottom of a Petri dish, which was modeled as a dielectric cylinder (35 mm diameter, 2 mm thickness) with a relative permittivity of 3.8. The set-up described was surrounded by a 70-mm diameter sphere of air.

A tetrahedral mesh chosen to discretize the domain of simulation resulted in a mesh element minimum size of 0.10 mm, a maximum size of 2.45 mm, and a total of 401,038 elements in a 22449.3 mm^3 volume of simulation. Quadratic elements were used throughout the solution domain, giving 0.54 x 10^6 degrees of freedom.

The Electric Currents interface was used to solve Maxwell's equations in steady-state conditions, for which:

$$\nabla \cdot (-\sigma \nabla V) = 0 \quad (1)$$

where V is the electric potential used to compute the electric field, $\boldsymbol{E}$=- ∇V, with the current density, $\boldsymbol{J}=\sigma\boldsymbol{E}$, where σ is the conductivity of the media. Under electrostatic conditions, the dispersive properties of the media were disregarded.

Quantification of nsEP polarity change

A stronger stimulation in the region where two bipolar waveforms overlapped into a unipolar pulse was observed consistently in all sets of experiments. The maximum effect at the focus of stimulation closely matched the effect of the first phase alone (Fig. 4F and S2, bottom panels) while the effect near the electrodes was attenuated by bipolar cancellation. The different waveforms formed by the superposition of bipolar nsEP from electrodes 2 and 3 (Fig. 3) provided varied degrees of cancellation which tapered from the edges to the center. We calculated the ratio (R) of the electric field vector amplitudes of the second and third phases to each phase that preceded it (i.e. E2/E1, E3/E1, and E3/E2; E values were taken from Fig. 3) to identify the relationship between the waveform shape and bipolar cancellation efficiency. The polarity ratios, R, were calculated as:

$$R1 = \frac{E_{2^{nd}}}{E_{1^{st}}} \qquad (2)$$

$$R2 = \frac{E_{3^{rd}}}{E_{1^{st}}} \qquad (3)$$

$$R3 = \frac{E_{3rd}}{E_{2nd}} \qquad (4)$$

Fig. S3A shows *R* values for 100/50/25%, 100/70/25%, and 100/70/40% nsEP conditions. A negative *R* indicates the two phases are opposite in polarity, while a positive *R* reveals the same polarity. The average of the absolute value of *R* (|*R*|), which indicates the extent of polarity change for each complex waveform, is plotted in Fig. S3B.

We found that each sequential increase in phase amplitude relative to the preceding phase resulted in an increase in the corresponding *R* near the electrodes. For example, with the 100/70/25% condition (Fig. S3A, middle panel), increasing the amplitude of the second phase to 70% from 50% increased E2/E1 near the electrodes, while E3/E1 and E3/E2 were the same or decreased, respectively. On the other hand, when both the second and third phase amplitudes were increased to 70% and 40%, respectively (Fig. S3A, right panel), all three calculated *R* values were increased at each electrode compared to the 100/50/25% condition. When we consider the average |*R*| (Fig. S3B), we find that the condition with the highest average |*R*| (100/70/40%) caused the most efficient cancellation from each bipolar nsEP (Fig. 5C, top panel). This corresponded to greater cancellation closer to each electrode and better stimulation targeting (Fig. 5C, bottom panel).

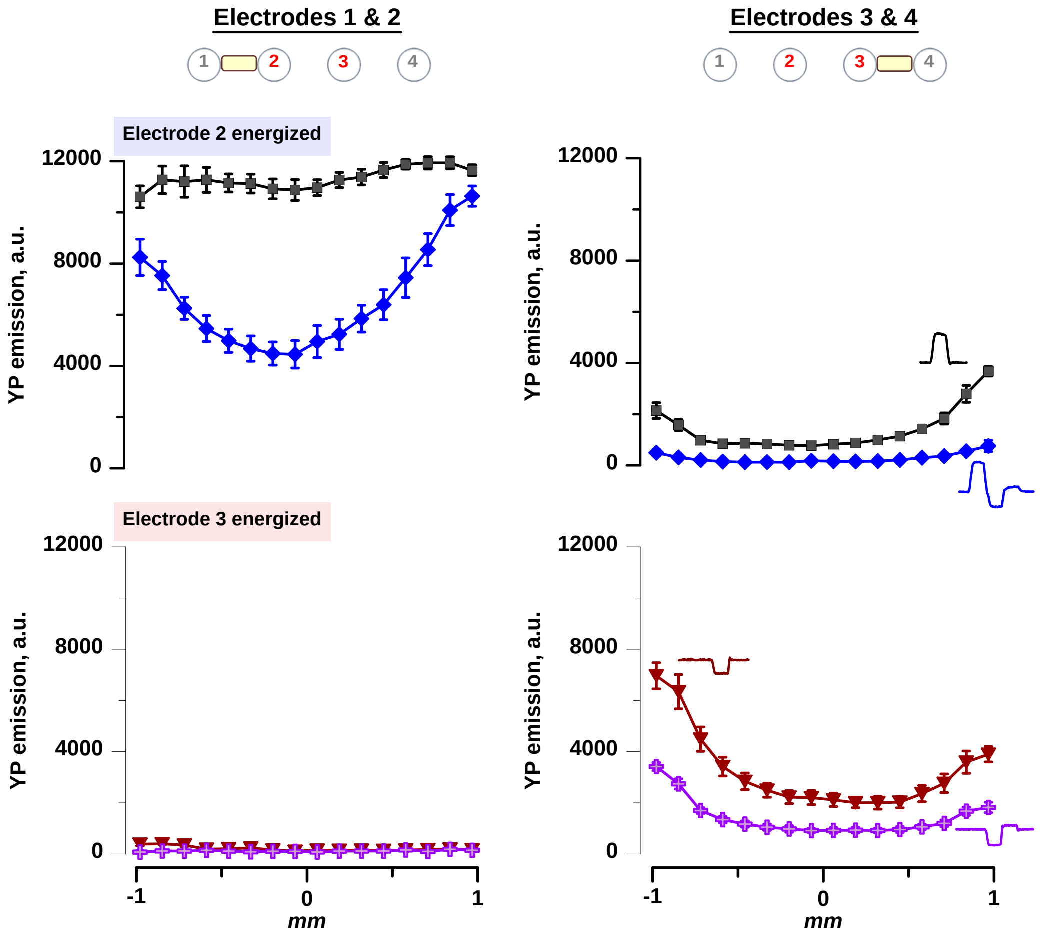

Fig. S1. Electropermeabilization effect from uni- or bipolar nsEP between electrode pairs 1 and 2 or 3 and 4. Stimulation effect was quantified by the uptake of YO-PRO-1 dye (YP) by electropermeabilized cells embedded in agarose gel. We delivered 100 stimuli at 10 Hz. When electrode 2 was energized (top panels), the nsEP phase amplitudes were 2.1, 1.1, and 0.4 kV (100/50/25%). The stimuli delivered when electrode 3 was energized (bottom panels) skipped the first phase and matched the second and third phase of the nsEP delivered from electrode 2. YP emission was measured in 16 regions of interest along a line between electrodes 1 and 2 (left panels) or 3 and 4 (right panels) and plotted against the distance from the center of the gap between the electrode pair (mean ± S.E., $n = 5$).

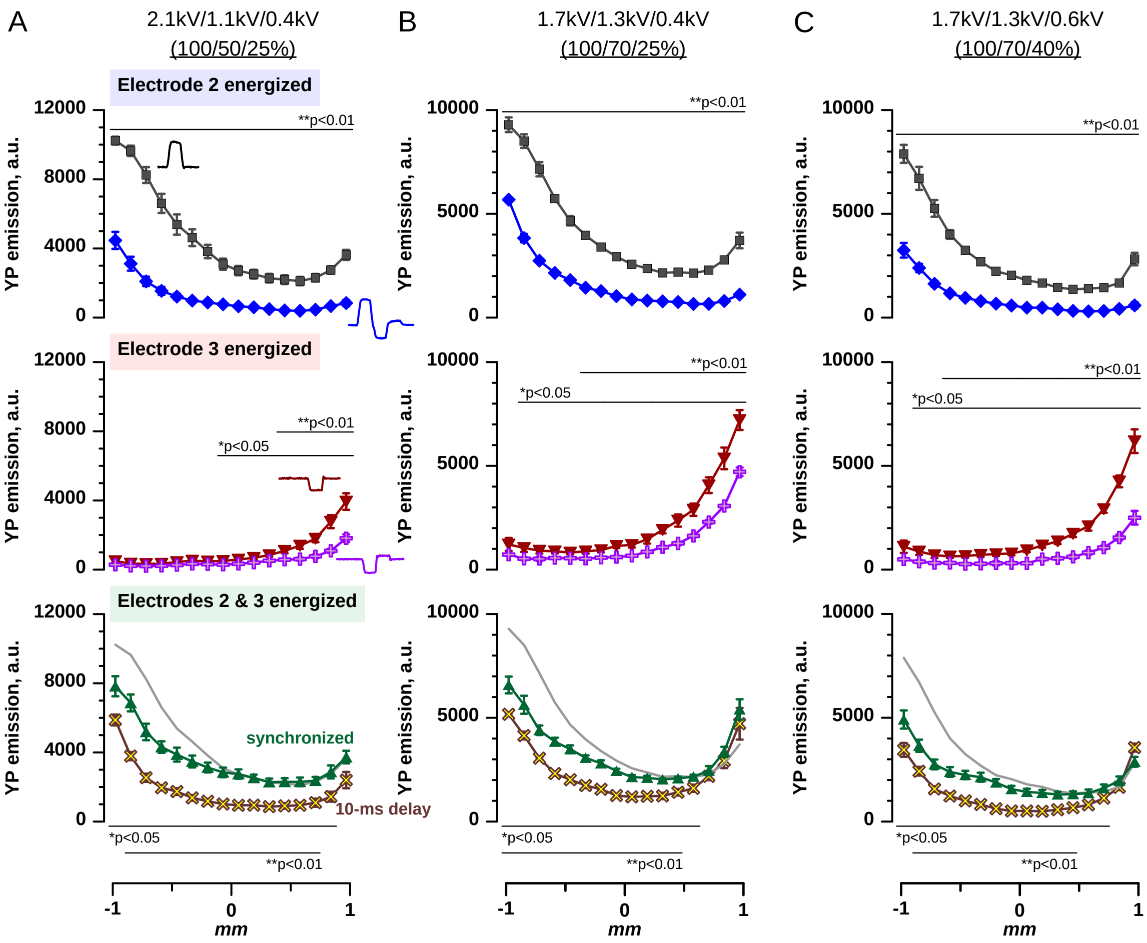

Fig. S2. Electropermeabilization effect from uni- or bipolar nsEP (with 2 or 3 phases). Cell electropermeabilization results from the experiments presented in Fig. 5. For panels **A-C**, the shapes of stimulating pulses and local waveforms arisen from their superposition are provided in Fig 3, **B-D**, respectively. The amplitude of each phase (kV) and their ratio (%) for a triphasic nsEP applied to electrode 2 are given at the top of each panel; biphasic pulses applied to electrode 3 are the same but omit the first phase. YP emission was measured following stimulation with uni- or triphasic nsEP from electrode 2 (top panels), uni- or biphasic nsEP from electrode 3 (middle panels), or triphasic and biphasic from electrodes 2 and 3, respectively, delivered either synchronously (with one phase shift) or with a 10-ms delay between them (bottom panels). Data are plotted against distance from the center of the gap between electrodes 2 and 3 along a line connecting them (mean ± S.E., n = 5-6). * p<0.5, ** p<0.01, two-tailed *t*-test.

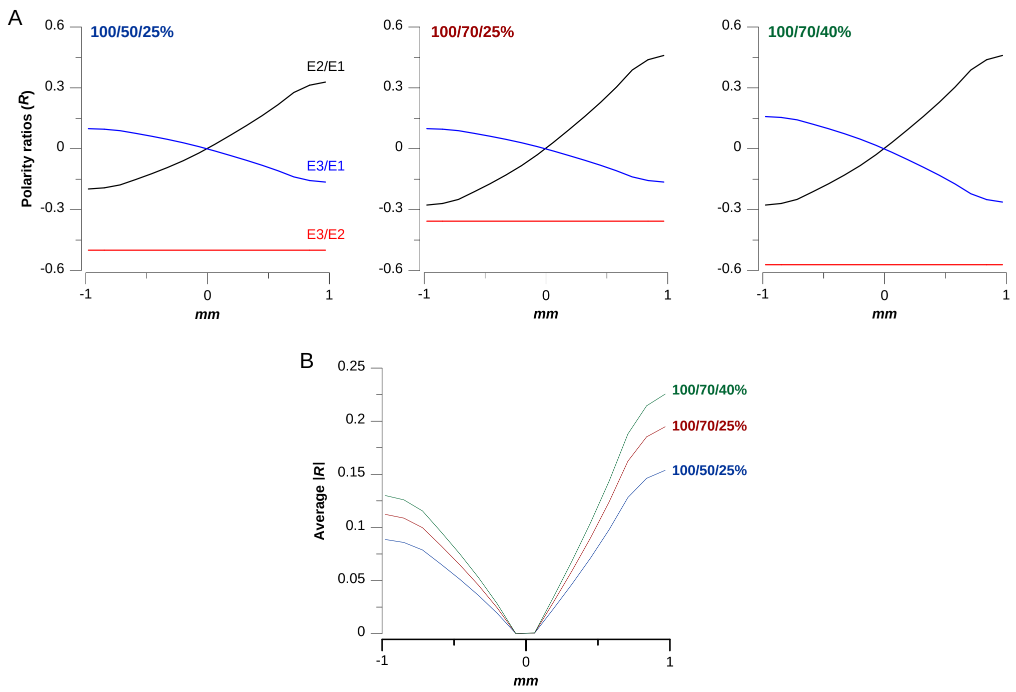

Fig. S3. Quantification of nsEP polarity change and bipolar cancellation effect. (**A**) The polarity ratio (*R*) of the E vector amplitudes (taken from Fig. 3) of the second to the first phase (E2/E1), the third to the first phase (E3/E1), and the third to the second phase (E3/E2) for each triphasic nsEP exposure condition (Equations 2-4, respectively) plotted as a function of distance between the electrodes. (**B**) The average of the absolute value of *R* (|*R*|) for each triphasic nsEP condition plotted as a function of distance. The greater the |*R*| value indicates a greater overall change in polarity (i.e. bipolarity). Notably, the condition with the highest |*R*| at the electrodes (100/70/40%) offered the most focused remote electroporation effect experimentally (see Fig. 5**C**).

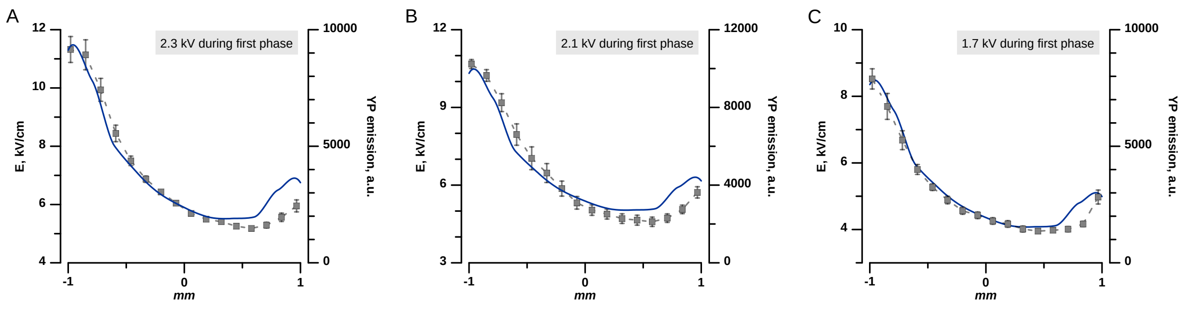

Fig. S4. Electropermeabilization effect follows the electric field distribution between electrodes 2 and 3. The electric field between electrodes 2 and 3 (solid blue line) was quantified for a first phase amplitude of 2.3 kV (**A**), 2.1 kV (**B**), or 1.7 kV (**C**) and plotted against distance from the center of the gap between the electrode pair. On the same plots, the YP emission caused by stimulation with unipolar nsEP delivered from electrode 2 with the same applied voltages are also shown (mean ± S.E., n = 5-6). The YP data in panels **A-C** are the same as that shown in Fig. 3**A**, S2**A**, and S2**C**, respectively.
